# Supplementary material for: Portable Sensors for Dynamic Exposure Assessments in Urban Environments: State of the Science
Source: Sensors (Basel). 2024 Aug 30;24(17):5653. doi: 10.3390/s24175653 (PMC11398000; doi:10.3390/s24175653)
Supplement: Supplementary file 1 [file sensors-24-05653-s001.zip › sensors-3068891-supplementary.pdf]

sensors

MDPI

Supplementary

Portable Sensors for Dynamic Exposure Assessments in Urban Environments: State of the Science

sensors

MDPI

Supplementary S1 Purchased portable sensor systems for the lab and field benchmarking study. *PT = prototype

Device	Manufacturer	Price class (€/unit)	Data Logging	GPS	Battery	Portability	Metrics	Resolution (sec)	Autonomy (h)
ATMOTube Pro	Atmotube	100-500	✓ Cloud	Smartphone	✓	Carabiner	TVOC, $PM_{1/2.5/10}$, P, Temp, RH	2 sec	24h
Airbeam 3	HabitatMap	100-500	✓ SD	✓	✓	Belt clip + carabiner	$PM_{1/2.5/10}$, Temp, RH	1 sec	17h
SODAQ AIR	SODAQ	100-500	✓ Cloud	✓	✓	Bicycle mount	$PM_{1/2.5/10}$	2sec – 5min	
SODAQ NO_2	SODAQ	PT*	✓ Cloud	✓	✓	No	$PM_{1/2.5/10}$, Temp, RH	2 sec – 5min	
PMScan	Tera	100-500	✓ Cloud	Smartphone	✓	Strap	$PM_{1/2.5/10}$, Temp, RH	1 sec	15h
PAM	2B Technologies	>1000	✓ Cloud	✓	✓	No	CO, CO2, $PM_{1/2.5/10}$, NO_2, Temp, Press, RH	2 sec	7h
ObservAir®	Distributed Sensing Technologies (DSTech)	>1000	✓ USB, SD, Cloud	✓	✓	No	BC, NO_2	2 sec	8h
GeoAir	Jaycon systems	PT*	✓ SD	✓	✓	Belt clip + fixation hole	PM_1, $PM_{2.5}$, PM_4, PM_{10}, tVOC, Temp, RH	1 sec (1 min)	12-15h
Open-Seneca	Open-Seneca	100-500	SD	✓	✓	Bicycle mount	PM_1, $PM_{2.5}$, PM_4, PM_{10}, Temp, RH	1 sec	5h
BCmeter	BCmeter	PT*	✓ SD	No	No	No	BC		No

sensors

MDPI

Supplementary S2 Obtained setpoint averages (μg/m³) for each sensor (1-3) and brand during the lack-of-fit testing for PM_1, $PM_{2.5}$, and PM_{10}.

	PM_1				$PM_{2.5}$				PM_{10}				
	PM_1_1	PM_1_2	PM_1_3	PM_1_REF	$PM_{2.5}$_1	$PM_{2.5}$_2	$PM_{2.5}$_3	$PM_{2.5}$_REF	PM_{10}_1	PM_{10}_2	PM_{10}_3	PM_{10}_REF	setpoints
ATMOTUBE	1.00	1.00	1.00	0.00	1.00	1.00	1.00	0.01	2.00	2.00	2.00	0.07	0
	2.13	2.00	2.00	2.51	6.27	5.60	5.87	8.90	10.47	9.33	9.60	30.56	30
	3.00	2.67	3.00	3.03	7.87	7.07	7.20	11.24	13.13	11.80	11.93	40.46	60
	7.00	6.47	6.47	6.03	17.33	16.00	15.80	25.82	29.47	26.40	26.13	102.33	110
	7.80	7.60	7.53	6.70	20.27	19.13	18.87	30.64	34.40	32.40	31.80	126.39	160
	17.47	16.20	17.00	13.98	43.07	39.73	40.27	63.38	73.13	66.47	66.53	248.61	250
	31.73	28.93	30.07	23.90	77.07	69.27	70.87	106.78	130.87	115.53	117.67	395.38	400
TERA PM SCAN	0.86	0.73	0.71	0.46	1.36	1.38	1.23	1.38	2.45	2.32	1.68	2.21	0
	3.26	3.24	2.84	2.09	5.01	5.83	5.11	7.17	9.89	11.07	10.79	23.08	30
	6.34	6.01	5.31	3.46	9.91	10.69	9.44	12.89	18.55	21.50	18.59	40.63	40
		8.93	8.09	4.84		16.13	15.60	19.07		30.69	31.05	61.58	60
		16.58	15.53	8.30		29.96	27.95	35.85		62.17	52.26	122.65	130
		21.86	20.24	10.49		39.61	38.47	47.50		82.59	75.54	166.28	200
	49.69	46.42	43.46	22.42	79.64	83.67	80.72	98.20	154.77	171.19	148.77	324.95	350
OPEN SENECA	0.25	0.20	0.18	0.21	0.38	0.30	0.28	0.26	0.54	0.42	0.39	0.42	0
	2.94	2.77	2.75	2.51	4.70	4.42	4.74	8.90	6.70	6.28	7.01	30.56	30
	3.49	3.25	3.36	3.03	6.21	5.57	5.98	11.24	9.34	8.23	9.01	40.46	60
	7.37	6.92	7.09	6.03	14.71	13.90	13.99	25.82	23.28	22.05	22.03	102.33	110
	8.16	7.80	8.04	6.70	16.77	16.20	16.08	30.64	26.86	26.05	25.48	126.39	160
	16.89	16.34	16.22	13.98	35.74	34.48	33.04	63.38	57.88	55.80	52.73	248.61	250
	29.75	28.06	28.66	23.90	62.96	59.43	59.15	106.78	101.97	96.29	94.90	395.38	400
SODAQ AIR	0.25	0.21	0.19	0.23	0.30	0.28	0.24	0.29	0.34	0.34	0.27	0.57	0
	3.84	3.18	2.30	2.51	8.71	5.84	4.55	8.78	14.45	8.94	7.19	29.62	30
	4.85	4.02	3.40	2.97	10.69	7.36	4.69	11.26	17.55	11.24	6.06	37.20	60
	10.58	8.08	6.48	6.11	25.64	18.47	12.33	26.22	43.49	30.82	19.15	104.66	110
	11.64	8.82	6.52	6.56	27.05	18.98	14.37	29.86	45.24	30.99	23.68	124.38	160
	23.22	17.47	13.32	13.75	57.60	41.16	28.70	62.38	98.41	69.40	46.91	250.92	250
	41.52	32.23	24.40	23.64	100.30	73.83	54.15	105.63	169.93	123.33	89.45	393.36	400
SODAQ NO2	0.04			0.00	0.07			0.01	0.11			0.13	0
	2.52			3.44	3.07			8.11	3.40			25.52	30
	2.34			3.58	2.82			8.95	3.09			27.79	40
	3.42			4.54	4.84			14.66	6.09			52.59	60
	4.68			5.96	7.31			22.26	9.84			86.73	110
	5.91			7.05	9.79			27.97	13.66			113.40	130
	12.42			14.48	23.50			61.67	35.18			229.12	250
	21.18			25.41	44.03			108.30	68.71			379.41	400
2BTECH PAM	0.20			0.00	0.20			0.01	0.20			0.08	0
	1.79			3.49	2.44			8.16	3.86			25.96	30
	1.93			3.65	2.37			8.84	4.18			28.18	40
	2.76			4.52	3.70			14.35	6.52			51.32	60
	4.75			6.01	7.04			22.59	10.90			89.03	110
	6.17			7.15	9.04			28.69	14.42			115.07	130
	8.55			14.34	17.34			60.87	29.06			228.86	250
	13.66			25.12	31.58			107.13	53.07			376.62	400

Supplementary S3 Obtained lack-of-fit curves and associated linear functions for each sensor (ATMO1-3, TERA1-3, OPEN1-3, AIR1-3 and NO_2_1-2; upper to lower) for the corresponding PM_1, $PM_{2.5}$, and PM_{10} particle size fractions (left to right). SODAQ NO_2_3 did not collect any data during the lack-of-fit test and is therefore not shown on the graph.

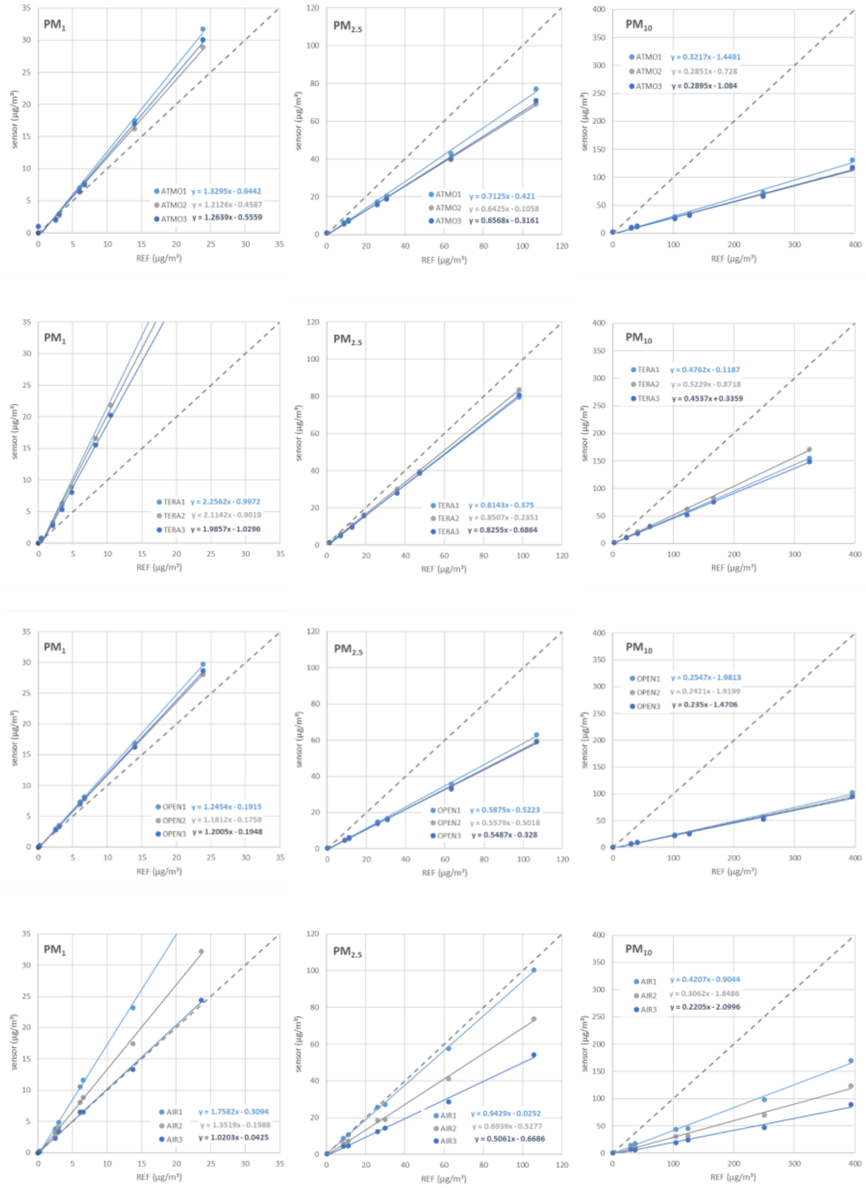

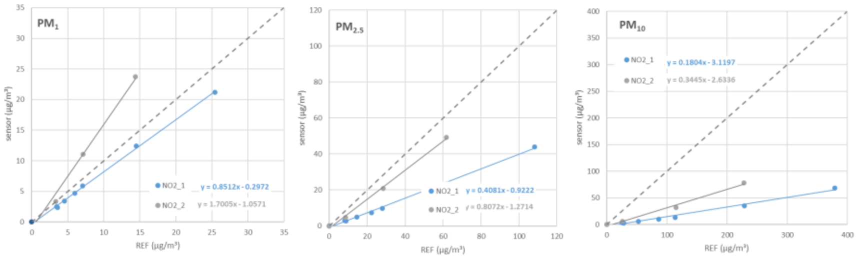

Supplementary S4 Coarse test results obtained on 14/7 (ATMOTUBE, OPEN SENECA, GeoAir, and SODAQ Air) and 2/9 (TERA, PAM, and SODAQ NO_2) with observed coarse composition (% coarse), PM_{10}, $PM_{2.5}$, and PM_{coarse} sensor/REF ratios, fine/coarse change ratio (%; between highlighted columns), and PM_{10} change ratio (%). *Faulty results due to peak mismatch.

	TEST	% COARSE	PM_{10} sensor/REF	$PM_{2.5}$ sensor/ REF	PM_{coarse} sensor/REF	%change in fine/coarse ratio (target=0)	%change in PM_{10} SENSOR/REF ratio (target=0)
**ATMOTUBE**	COARSE (7750nm)	75	0.02	0.05	0.02	**37***	**-19***
	FINE (1180nm)	14	0.02	0.01	0.07		
**OPEN SENECA**	COARSE (7750nm)	75	0.16	0.38	0.09	**-76**	**72**
	FINE (1180nm)	14	0.58	0.38	1.78		
**SODAQ Air**	COARSE (7750nm)	75	0.17	0.37	0.10	**-73**	**72**
	FINE (1180nm)	14	0.62	0.39	2.00		
**GeoAir**	COARSE (7750nm)	75	0.21	0.50	0.11	**-76**	**67**
	FINE (1180nm)	14	0.63	0.44	1.76		
**TERA**	COARSE (7750nm)	97	0.05	0.73	0.03	**-94**	**93**
	FINE (1180nm)	27	0.72	0.49	1.33		
**SODAQ NO_2**	COARSE (7750nm)	97	0.01	0.18	0.00	**-99**	**97**
	FINE (1180nm)	27	0.23	0.18	0.35		
**PAM**	COARSE (7750nm)	97	0.00	0.13	0.00	**-100**	**97**
	FINE (1180nm)	27	0.13	0.08	0.26		

Supplementary S5 Obtained setpoint averages for the considered NO_2 sensor systems.

	SENSOR 1					SENSOR2					SENSOR 3				
	NO2_sensor	NO2_REF	SD_sensor	SD_REF	setpoints	NO2_sensor	NO2_REF	SD_sensor	SD_REF	setpoints	NO2_sensor	NO2_REF	SD_sensor	SD_REF	setpoints
SODAQ NO2					0	-18.4	-0.02	60.27	0.02	0	-2	-0.04	46.22	0.03	0
					40	-66.13	49.55	107.82	0.29	40	-45.57	49.62	110.03	0.27	40
	-325	111.81	5.29	0.2	100	-156.36	111.75	63.88	0.22	100	-145.78	111.7	51.49	0.16	100
					140	-180.13	136.29	41.1	0.23	140	-150.62	136.29	104.92	0.2	140
	-437	201.98			200	-238.9	202.05	64	0.36	200	-242.36	202.17	85.04	0.47	200
			**5.29**	**0.2**	**Stability (SD)**			**67.42**	**0.23**	**Stability (SD)**			**79.54**	**0.22**	**Stability (SD)**
PAM	**NO2_sensor**	**NO2_REF**	**SD_sensor**	**SD _REF**	**setpoints**										
	-9.72	-0.02	35.23	0.02	0										
	20.98	49.61	31.84	0.19	40										
	81.83	111.69	26.31	0.24	100										
	103.57	136.38	19.44	0.19	140										
	212.89	202.07	21.61	0.28	200										
			**26.89**	**0.19**	**Stability (SD)**										
Observair	**NO2_sensor**	**NO2_REF**	**SD_sensor**	**SD_REF**	**setpoints**										
	0.02	-0.02	<0.01	0.03	0										
	0.01	49.63	<0.01	0.23	40										
	0	111.71	<0.01	0.24	100										
	-0.01	136.36	<0.01	0.21	140										
	-0.02	202.07	<0.01	0.31	200										
			**<0.01**	**0.2**	**Stability (SD)**										

Supplementary S6 Obtained lack-of-fit curves and associated linear functions for each sensor system; SODAQ NO_2 (1-3), PAM, and Observair.

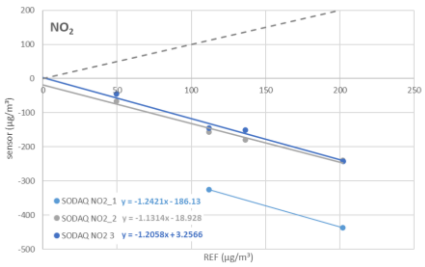

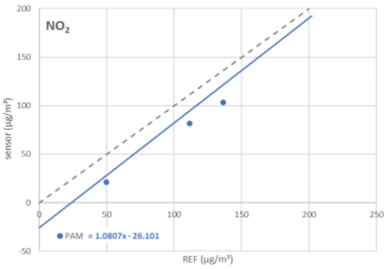

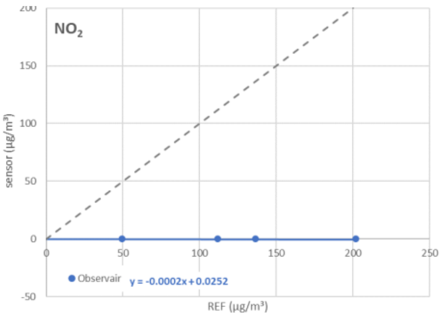

Supplementary S7 Lab-calibrated NO_2 sensor response to varying relative humidity steps (0-90-75-50-0%) under zero (upper) and span (lower) concentrations.

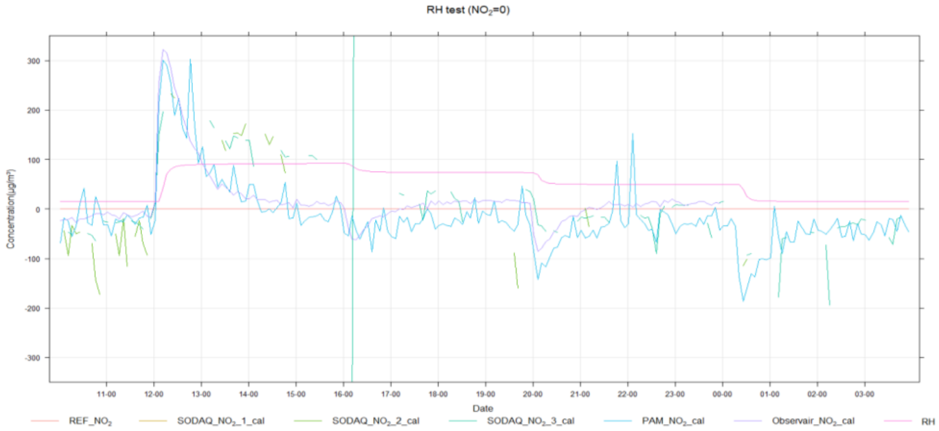

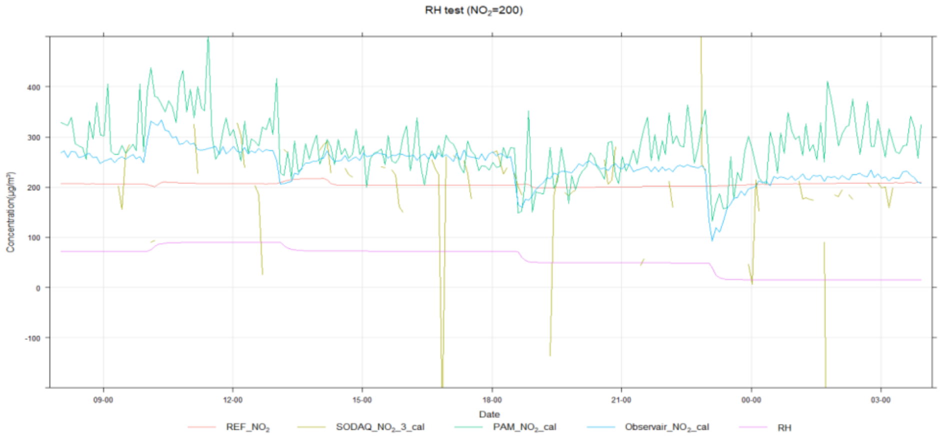

Supplementary S8 Lab-calibrated NO_2 sensor response to varying temperature steps (-5, 10, 20 and 30°C) under zero (upper) and span (lower) concentrations.

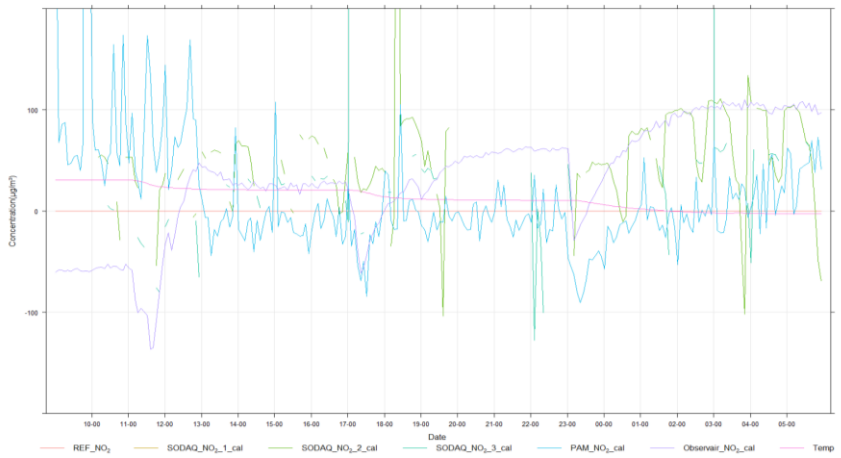

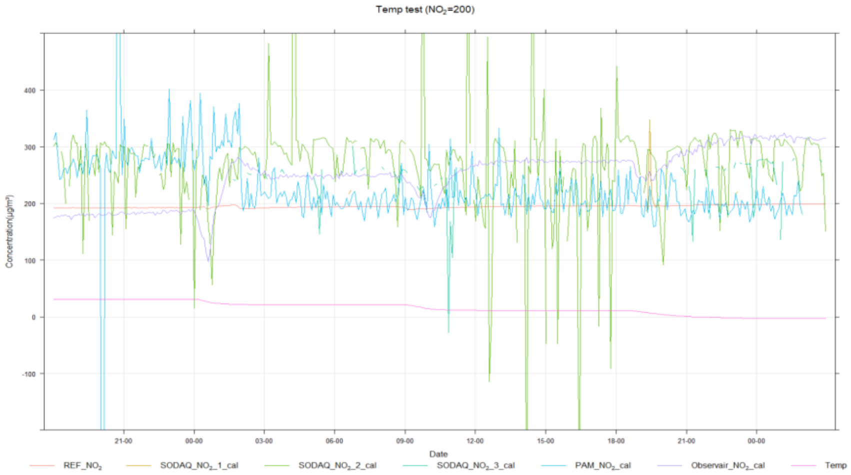

Supplementary S9 Response test setup, NO_2 average (AVG), 90-percentile (90%) concentration and associated response time (t_90), calculated for the Observair and PAM sensor systems and Thermo NOx analyzer

		AVG	90%	t_90
**Plateau 1**	Thermo	215	193	3 min
	Observair_cal	295	265	2 min
	PAM_cal	197	177	3 min
**Plateau 2**	Thermo	216	194	3 min
	Observair_cal	286	258	1-2 min
	PAM_cal	177	159	1 min
**Plateau 3**	Thermo	217	195	3 min
	Observair_cal	291	262	1 min
	PAM_cal	175	158	2 min

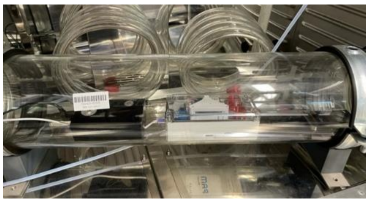

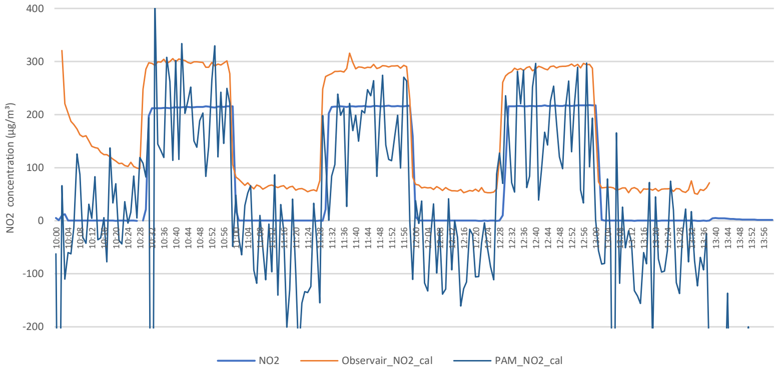

Supplementary S10 Observed $PM_{2.5}$ (left) and BC (right) concentrations experienced by, respectively, the Grimm 11D and Aethlabs MA200 during the mobile field test.

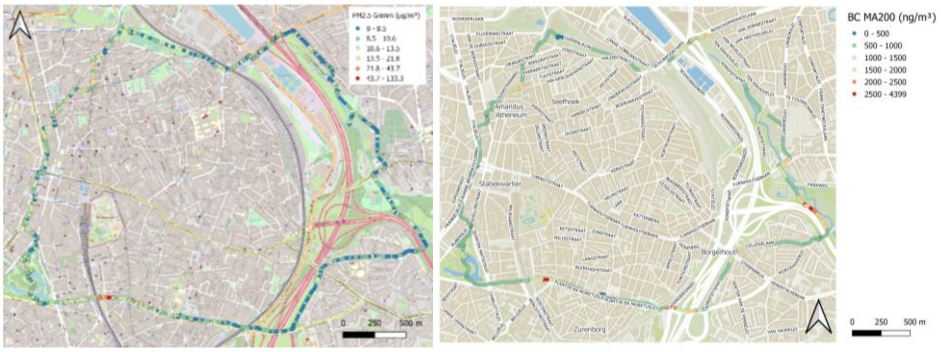

Commented [EE1]: Attention: Second S9. Legend not in manuscript document.

Commented [JH2R1]: Changed to S10 and added in manuscript

Supplementary S11 Average horizontal accuracy (m) and number of datapoints (n) of the considered sensor systems during the mobile field test.

	Horizontal distance to REF track (m)	Count (n)
TomTom (REF)	0.00	6077
TERA PMscan	2.28	1008
OPEN SENECA	3.18	489
SODAQ NO2	3.73	78
2BTech PAM	4.20	2476
SODAQ AIR	4.28	449
ATMOTube Pro	5.43	4
DST Observair	7.35	2446
GeoAir	8.15	4616

Supplementary S12 Black carbon (μg/m³) concentration maps generated from the mobile measurements conducted by the Aethlabs MA200 and Observair during the mobile field test in Antwerp, Belgium.

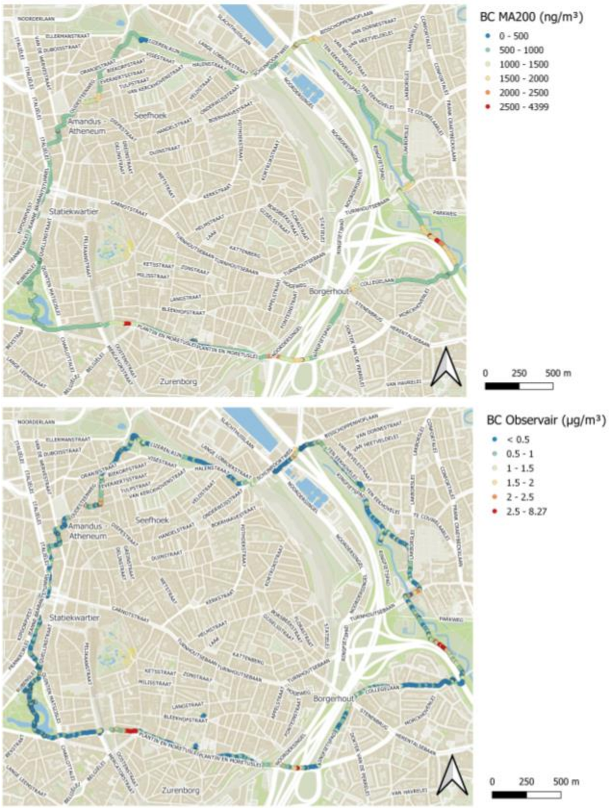

Supplementary S13 Temporal pollutant variability of PM, BC, NO_2, and O_3 at R801 during the field co-location campaign. Shadings denote 95% confidence intervals.

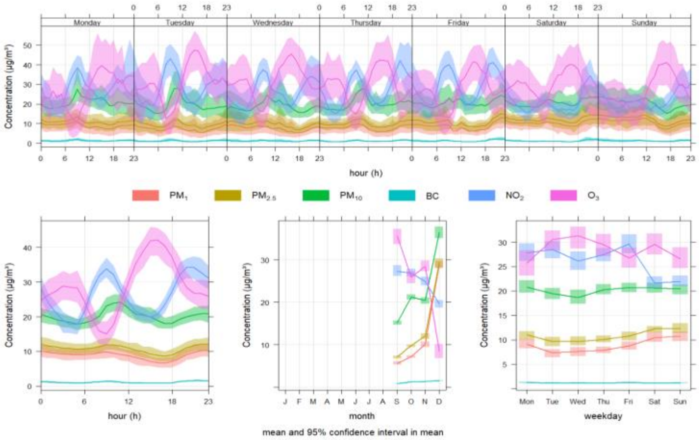

Supplementary S14 Sensitivity of the considered PM sensor systems towards relative humidity (%) as observed during the field campaign by elevated sensor/REF $PM_{2.5}$ ratios under increasing relative humidity (%). Mind the different ranges in relative humidity between the sensors, resulting from the varying data availabilities for some of the sensor systems (explained in §3.3).

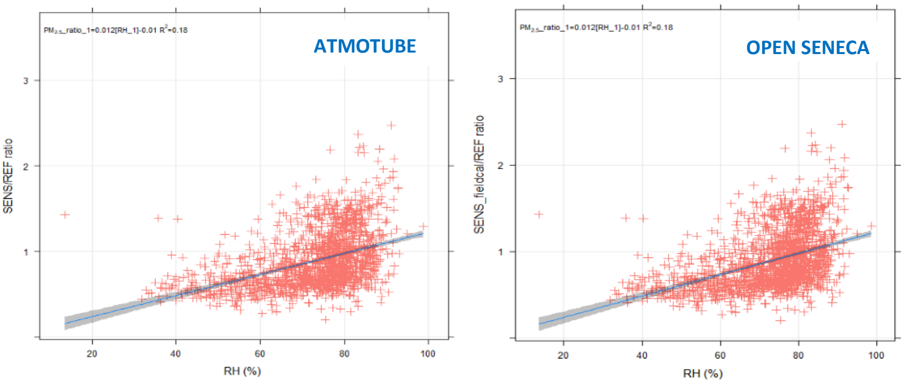

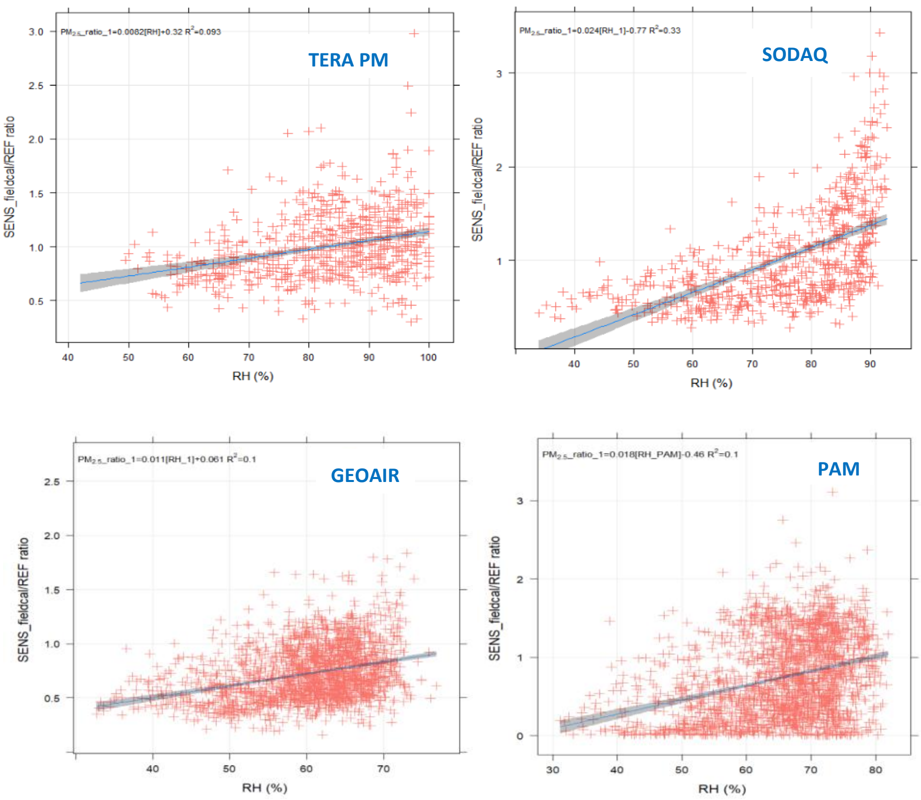
PM2.5_ratio_1=0.0082[RH]+0.32 R2=0.093
TERA PM
SENS_fieldcal/REF ratio
RH (%)
PM2.5_ratio_1=0.024[RH_1]-0.77 R2=0.33
SODAQ
SENS_fieldcal/REF ratio
RH (%)
PM2.5_ratio_1=0.011[RH_1]+0.061 R2=0.1
GEOAIR
SENS_fieldcal/REF ratio
RH (%)
PM2.5_ratio_1=0.018[RH_PAM]-0.46 R2=0.1
PAM
SENS_fieldcal/REF ratio
RH (%)
